# Supplementary material for: Causal Relationship between Aging and Anorexia Nervosa: A White-Matter-Microstructure-Mediated Mendelian Randomization Analysis
Source: Biomedicines. 2024 Aug 16;12(8):1874. doi: 10.3390/biomedicines12081874 (PMC11351342; doi:10.3390/biomedicines12081874)
Supplement: Supplementary file 1 [file biomedicines-12-01874-s001.zip › Supplementary Figure/Figure S8/Leaveoneout plot of MR analysis for TL to ubm-b-1945.pdf]

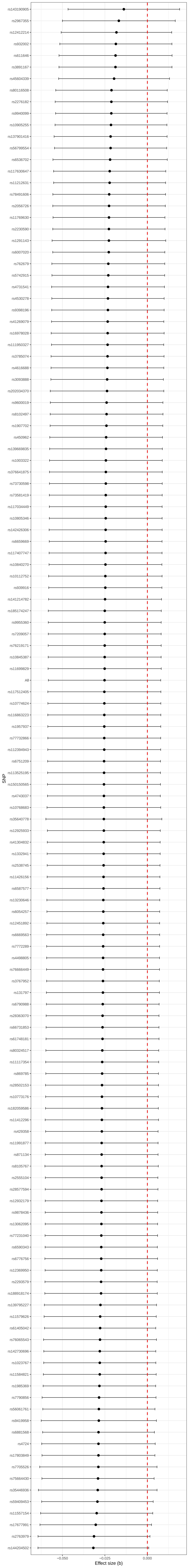
rs143190905
rs2967355
rs12412214
rs932002
rs611646
rs3891167
rs45604339
rs80116508
rs2276182
rs9940099
rs10905255
rs137901416
rs56799554
rs6536702
rs117630647
rs11212631
rs78491606
rs2056726
rs11769630
rs2230590
rs1291143
rs6007020
rs762679
rs5742915
rs4731541
rs4530278
rs9398196
rs41269079
rs16978028
rs111950327
rs3785074
rs4616688
rs3093888
rs202034370
rs9600019
rs8102497
rs1907702
rs450962
rs139669835
rs1003322
rs376641875
rs73730598
rs73581419
rs117034449
rs10805346
rs142426306
rs6659669
rs117407747
rs10840270
rs10112752
rs939916
rs141214782
rs185174247
rs9955360
rs7209057
rs76219171
rs10845387
rs11699829
All
rs117512405
rs10774624
rs116863223
rs1957937
rs77732866
rs112394943
rs6751209
rs113525195
rs150150565
rs4743037
rs10768683
rs35640778
rs12925933
rs41304832
rs1332941
rs2538745
rs11426156
rs6587577
rs13230646
rs6054257
rs12451892
rs6669563
rs7772289
rs4498805
rs76666449
rs3767952
rs131797
rs6790988
rs28363070
rs66731853
rs61748181
rs80324517
rs11117354
rs869785
rs28502153
rs10773176
rs182059586
rs11412296
rs429358
rs11991877
rs871134
rs8105767
rs2555104
rs28577594
rs12932179
rs9878436
rs13062095
rs77231040
rs6590343
rs6776756
rs12369950
rs2293579
rs188918174
rs139795227
rs11579626
rs61405042
rs76065543
rs142730696
rs1023767
rs11584821
rs1985369
rs7790856
rs56061761
rs9419958
rs6881568
rs4724
rs17803849
rs7705526
rs75664430
rs35446936
rs59409453
rs11557154
rs17677991
rs2763979
rs144204502
SNP
−0.050
−0.025
0.000
Effect size (b)
